# *Calixomeria*, a new genus of Sceliotrachelinae (Hymenoptera, Platygastridae) from Australia

**DOI:** 10.3897/zookeys.830.32463

**Published:** 2019-03-14

**Authors:** Zachary Lahey, Lubomír asner, Norman F. Johnson

**Affiliations:** 1 Department of Evolution, Ecology, and Organismal Biology, The Ohio State University, 1315 Kinnear Road, Columbus, Ohio 43212, USA The Ohio State University Columbus United States of America; 2 Agriculture and Agri-Food Canada, K.W. Neatby Building, Ottawa, Ontario K1A 0C6, Canada Agriculture and Agri-Food Canada Ottawa Canada

**Keywords:** Parasitoid, Platygastroidea, taxonomy

## Abstract

*Calixomerialasallei***gen. n. et sp. n.** is described as a new genus and species of Sceliotrachelinae. *Calixomeria* most closely resembles genera of the *Aphanomerus*-cluster but possesses several characters that readily separate it from other sceliotracheline genera. The key of Masner and Huggert (1989) is modified to accommodate *Calixomeria*, and the relationship of the genus to other members of the subfamily is discussed.

## Introduction

Platygastroidea is well represented in Australia. Approximately 10% (740 species in 80 genera) of all described species occur there, with an estimated 1800 species left to be described (ABRS 2015). A disproportionate amount of that diversity, however, has been described in the family Scelionidae, one of two families that classically comprise the superfamily Platygastroidea (Talamas and Buffington 2015). Much less attention has been paid to Platygastridae, and even less to the subfamily Sceliotrachelinae, due to their small size (most species < 1 mm) and rarity in collections (Masner and Huggert 1989).

The first Australian sceliotrachelines were described by Robert C. L. Perkins during his search for natural enemies of leafhoppers as an entomologist with the Hawaiian Sugar Planters’ Association (Perkins 1905). Shortly thereafter, Alan P. Dodd, then an assistant entomologist with the Bureau of Sugar Experiment Stations, added two genera, *Aphanomerella* Dodd and *Platygastoides* Dodd (Dodd 1913a, b). Following Dodd, descriptions of Australian sceliotrachelines all but stopped until the landmark work of Masner and Huggert (1989), who erected 13 new genera, including several known only from Australasia. The purpose of our research is to follow in the footsteps of Perkins and Dodd by describing an unusual new genus of Sceliotrachelinae from southern Australia.

The contributions of the authors are as follows: Z. Lahey: character definition and coding, generic concept development, species concept development, imaging, key development, manuscript preparation; L. Masner and N. F. Johnson: character definition, generic concept development, species concept development.

## Materials and methods

The numbers prefixed with “OSUC” or “USNMENT” are unique identifiers for the individual specimens (note the blank space after some acronyms). Details of the data associated with these specimens may be accessed at the following link: https://hol.osu.edu, and entering the identifier in the form.

Abbreviations and morphological terms used in the text: sensillar formula of clavomeres: distribution of the large papillary sensilla on the ventral clavomeres of the adult female (Bin et al. 1989; Yang et al. 2016), with the segment interval specified followed by the number of papillary sensilla (PS) per segment (e.g., A10–A8/1-2-2) (Bin 1981); T1, T2, ... T6: metasomal tergite 1, 2, ... 6; S1, S2, … S6: metasomal sternite 1, 2, … 6. Morphological terminology generally follows Masner and Huggert (1989), Mikó et al. (2007), and Talamas and Masner (2016). Morphological terms were matched to concepts in the Hymenoptera Anatomy Ontology (Yoder et al. 2010) using the text analyzer function.

Images were captured with a Leica MC170 HD digital camera attached to a Leica Z16 APOA microscope using Leica Application Suite (version 4.12.0), or with a Canon EOS 70D attached to an Olympus BX51. Image stacks were combined into a single montage image using Zerene Stacker (version 1.04). Montage images were postprocessed with Adobe Photoshop CS6 Extended and are archived at https://specimage.osu.edu, the image database at The Ohio State University.

Scanning electron micrographs were produced with a Thermo Fisher Scientific Apreo Scanning Electron Microscope. The specimen was disarticulated with a minuten probe on a 0.5-inch slotted aluminum mounting stub using carbon adhesive tabs. The specimen was not coated.

### Collections

This work is based on specimens deposited in the following repositories:

**ANIC** Australian National Insect Collection, Canberra, ACT, Australia

**CNCI** Canadian National Collection of Insects, Ottawa, Ontario, Canada

**OSUC** C.A. Triplehorn Collection, The Ohio State University, Columbus, Ohio, USA

**USNM** National Museum of Natural History, Washington, DC, USA

**Abbreviations and characters annotated in the figures**:

**apT2** anterior setal patch on T2 (Fig. 19)

**atp** anterior tentorial pit (Fig. 9)

**auc** axillular carina (Fig. 12)

**ax** axilla (Fig. 12)

**axu** axillula (Fig. 12)

**cly** clypeus (Fig. 9)

**Cu** cubital vein (Fig. 17)

**fed** femoral depression (Fig. 13)

**fp** foamy structures on propodeum (Fig. 19)

**mkT1** median keel on T1 (Fig. 19)

**lpa** lateral pronotal area (Fig. 14)

**lpc** lateral propodeal carina (Fig. 22)

**M+Cu** fusion of medial and cubital veins (Fig. 17)

**metp** metapleural pit (Fig. 13)

**mgps** multiporous grooved peg sensillum (Figs 6, 7)

**mnt** metanotal trough (Figs 15, 16)

**msc** mesoscutum (Fig. 11)

**mtpc** metapleural carina (Fig. 15)

**mtps** metapleural sulcus (Fig. 13)

**pxcs** paracoxal sulcus (Fig. 13)

**ppd** propodeum (Fig. 15)

**prcs** pronotal cervical sulcus (Fig. 13)

**ps** papillary sensillum (Figs 6, 8)

**psu** posterior mesoscutellar sulcus (Fig. 20)

**R** submarginal vein (Fig. 17)

**RS+M** basal vein (Fig. 17)

**scu** mesoscutellum (Fig. 11)

**sss** scutoscutellar sulcus (Figs 11, 12)

**tel** transepisternal line (Fig. 21)

**tsa** transcutal articulation (Fig. 12)

## Taxonomy

### Calixomeria lasallei

Taxon classificationAnimaliaHymenopteraPlatygastridae

Lahey & Masner, gen. n. et sp. n.

http://zoobank.org/B02021B7-DBBB-4C18-8231-42B6C09C6033

http://zoobank.org/55B75102-179B-4F0D-9376-DB5E8F6013A7

Figures 1–4 , 5–10 , 11–16 , 17–18

#### Description.

Body length 0.71–0.85 mm (*n* = 20). Squat, dorsoventrally flattened.

***Head*.** Color of head: light to dark brown. Shape of head in anterior view: nearly triangular. Shape of head in dorsal view: ovoid to semicircular. Shape of vertex: flat anteriorly, sharply angled posteriorly. Setation of compound eye: present. Occipital pit: absent. Position of lateral ocellus: remote from inner orbit, OOL > 3 ocellar diameters. Length of LOL: equal to OOL. Shape of frons: sharply angled anterior to anterior ocellus. Sculpture of gena: alutaceous. Shape of gena: strongly receding behind compound eye. Median sulcus of postgenal bridge: setose. Malar sulcus: absent. Epistomal sulcus: absent. Shape of clypeus: convex. Anteclypeus: undifferentiated. Orientation of mandibular teeth: transverse. Mandibular dentition: bidentate. Number of maxillary palpomeres: 1. Number of labial palpomeres: 1. Number of antennomeres: 10. A7: fused to clavomere A8. Shape of A7: 1.5× as wide as long, distinctly wider and longer than A6. Number of clavomeres: 3. Sensillar formula of clavomeres: A10–A8/1-2-2.

***Mesosoma*.** Color of mesosoma: light to dark brown. Epomium: absent. Lateral pronotal area: strongly excavate below anterior margin of pronotal shoulders. Form of pronotal cervical sulcus: indicated as narrow groove dorsally. Setation of pronotal cervical sulcus: absent. Sculpture of pronotal shoulders: imbricate. Pronotal shoulders: visible in dorsal view. Anterior margin of mesoscutum: not reflexed, on same plane as posterior margin of pronotum. Sculpture of mesoscutum: imbricate. Shape of mesoscutum: pentagonal, curved along anterior margin. Anterior admedian line: absent. Median mesoscutal line: absent. Notaulus: absent. Parapsidal line: absent; present. Netrion: absent. Axilla: present, almost hidden in dorsal view. Sculpture of mesoscutellum: imbricate. Length of mesoscutellum: nearly equal to maximum width. Shape of mesoscutellum: semielliptical. Metascutellum: weakly carinate medially, undifferentiated from metanotal trough. Sculpture of metanotal trough: smooth. Sculpture of mesopleuron posterior to femoral depression: transversely striate. Sculpture of femoral depression: sometimes with faint traces of transverse striation. Sculpture of ventral mesopleuron: reticulate. Mesofemoral depression: present. Mesopleural carina: absent. Metapleural carina: present. Metapleural pit: present, located at anterior margin; Paracoxal sulcus: present as a smooth furrow below metapleural pit. Sculpture of propodeum: mostly smooth, weakly carinate medially, weakly rugose anterolaterally. Shape of legs: laterally compressed, especially hind coxae. Protibial spur: simple, curved, without comb. Tibial spur formula: 1-1-1. Tarsal formula: 5-5-5.

***Metasoma*.** Color of metasoma: light to dark brown. Shape of metasoma: distinctly longer than wide, narrowed apically. Number of visible terga: 6. Number of visible sterna: 6. Sculpture of T1: mostly smooth, weakly carinate along anterior margin. Sculpture of terga: T2–T5 weakly reticulate laterally, smooth medially. Setation of terga: present. Shape of setae on terga: stout, straight. Number of setae on terga: increasing in number from T2–T5. Setation of T2–T4: present laterally, absent medially. Setation of T5–T6: present across tergite. Sculpture of sterna: not apparent. Laterotergites: present. Sculpture of laterotergites: absent. Setation of laterotergites: present. Laterosternites: absent. Shape of T1: trapezoidal, widening posteriorly. Longest tergite: T2, 2.5× as long as T3. Transverse furrow on anterior margin of T2: present. Shape of T6: triangular. Transverse felt field on anterior S2: absent. Pilosity of S2: dense.

***Wings*.** Wing development: macropterous. Length of fore wing: extending to apex of metasoma. Marginal cilia of fore wing: present, longest along apical margin. Color of wings: hyaline basally, fuscous distally. Length of fore wing submarginal vein: 1/3 to greater than 1/2 fore wing length. Submarginal vein of fore wing: tubular basally, gradually becoming a tessellated line of cells medially, terminating in a nebulous knob. Shape of fore wing submarginal vein: straight. Shape of knob of submarginal vein: circular, with a single spine-like seta emerging from anterodorsal margin. Basal vein of fore wing: nebulous. Cubital vein of fore wing: nebulous basally, weaker distally. Marginal cilia of hind wing: present, longest along posteroapical margin. Submarginal vein of hind wing: present.

***Male*.** Unknown.

***Biology*.** Unknown.

**Figures 1–4.**
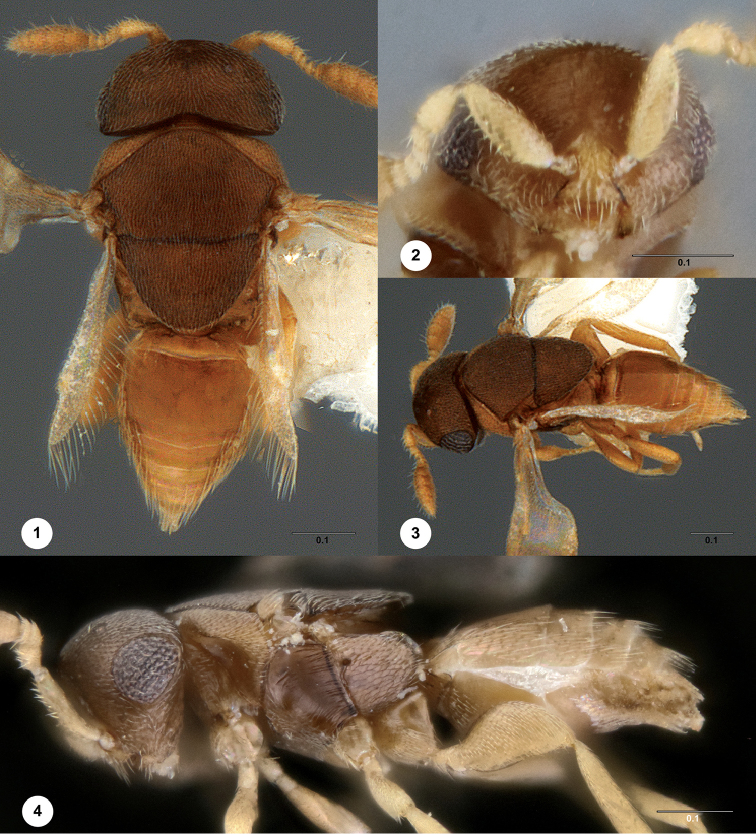
*Calixomerialasallei***1** female holotype (OSUC 711133), head, mesosoma, metasoma, dorsal view **2** female (USNMENT01197947), head, anterior view **3** female holotype (OSUC 711133), head, mesosoma, metasoma, dorsolateral view **4** female (USNMENT01197947), head, mesosoma, metasoma, lateral view. Scale bar: in millimeters.

**Figures 5–10.**
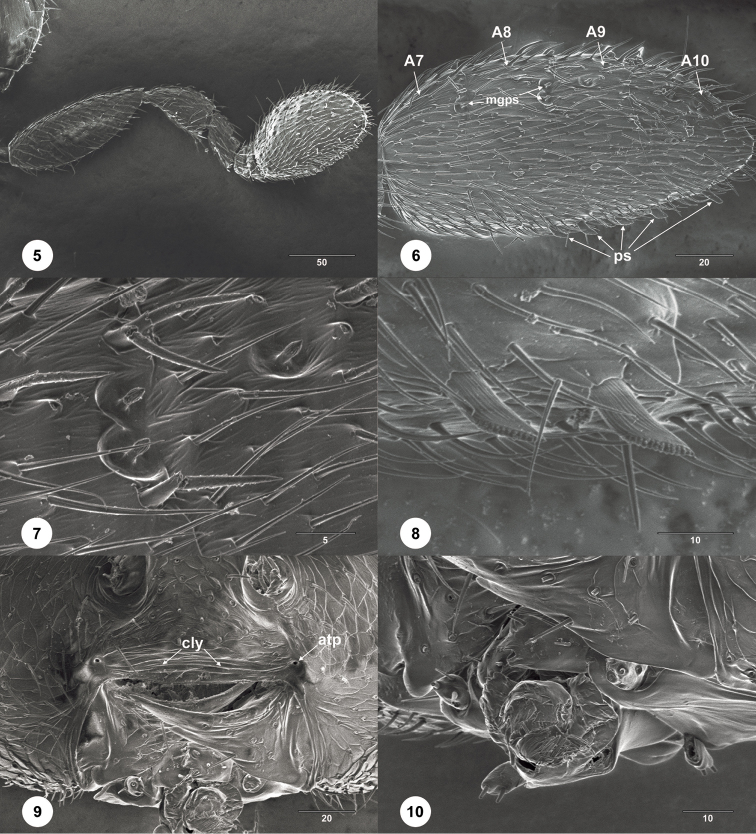
*Calixomerialasallei* female (USNMENT01197947) **5** antenna, lateral view **6** clavomeres, lateral view **7** multiporous grooved peg sensilla, dorsal view **8** papillary sensilla, dorsolateral view **9** mouthparts, anterior view **10** maxillary and labial palps, anterolateral view. Scale bar: in micrometers.

**Figures 11–16.**
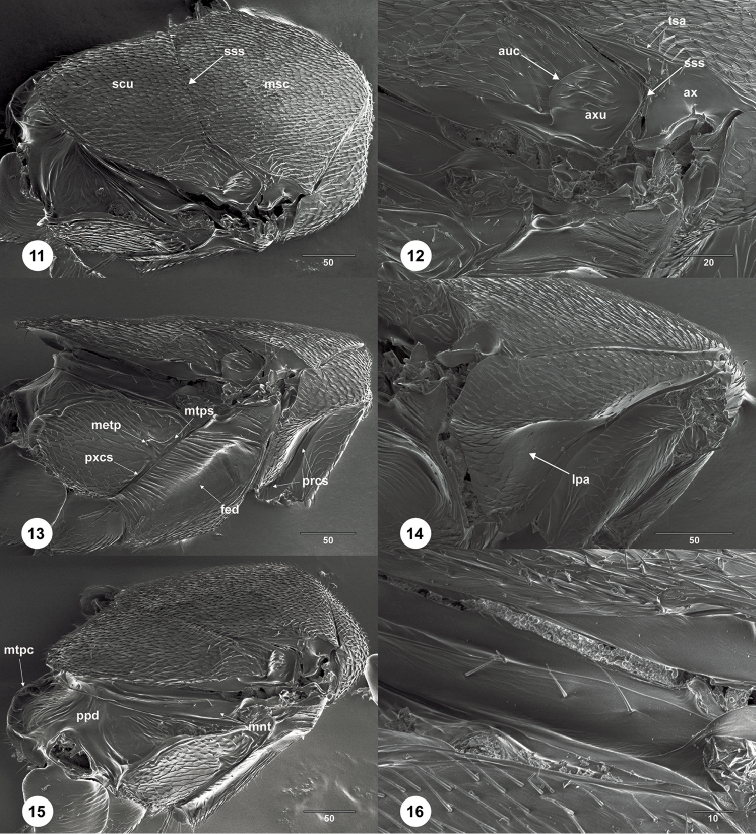
*Calixomerialasallei* female (USNMENT01197947) **11** mesosoma, dorsolateral view **12** axillar complex, dorsolateral view **13** mesosoma, lateral view **14** pronotum, anterolateral view **15** mesosoma, posterodorsal view **16** metanotal trough, posterodorsal view. Scale bar: in micrometers.

**Figures 17, 18.**
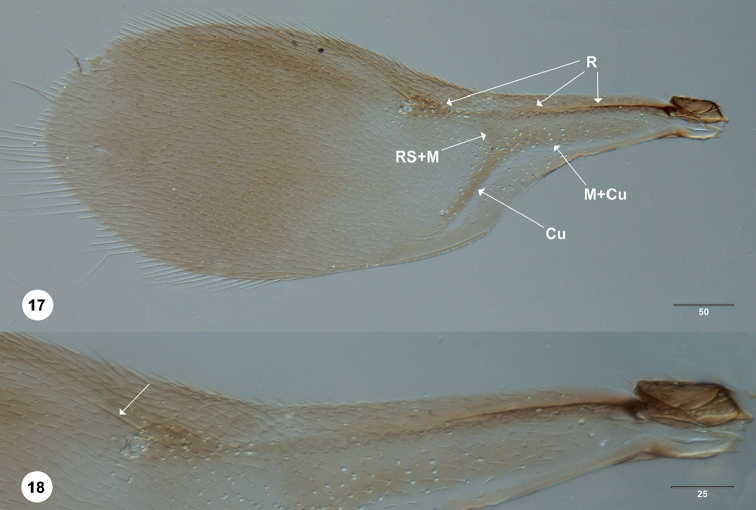
*Calixomerialasallei* female (OSUC 711154) **17** fore wing, dorsal view **18** close-up of R vein, with the arrow indicating the elongate seta emerging from the knob, dorsal view. Scale bar: in micrometers.

**Figures 19–22.**
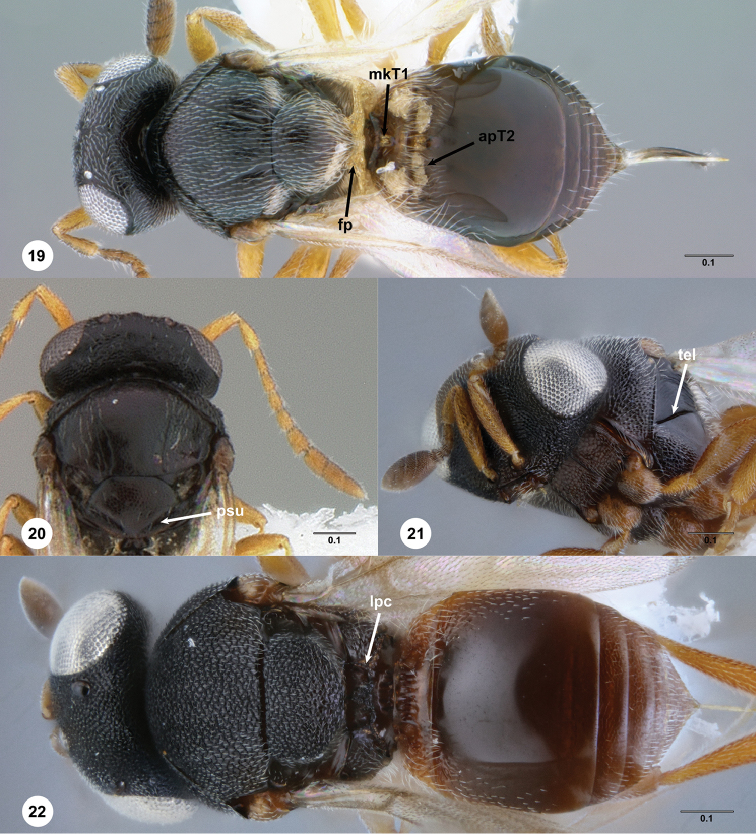
**19***Helavaaureipes* Masner & Talamas female holotype (USNMENT00989205), head, mesosoma, metasoma, dorsal view **20***Alfredella* sp. female (USNMENT00916677), head, mesosoma, dorsal view **21***Aphanomerus* sp. female (USNMENT01109890), head, mesosoma, ventrolateral view **22***Aphanomerus* sp. female (USNMENT01109890), head, mesosoma, metasoma, dorsal view. Scale bar: in millimeters.

#### Etymology.

The genus name is derived from the Latin word for ‘cup’ (*calix*) in reference to the shape of the mesoscutellum in dorsal view. The gender is feminine. This species is named in memory of Dr John La Salle for his lifetime of achievements that have advanced our knowledge of the parasitic Hymenoptera and biodiversity of Australia.

#### Link to Distribution Map.

[http://hol.osu.edu/map-large.html?id=410778]

#### Material examined.

Holotype, female: **AUSTRALIA**: ACT, Blundells Creek, 35°22'S, 148°50'E, 850 m, 3 km E Piccadilly Circus, March 1985, flight intercept trap/window trough trap, Lawrence, Weir, & Johnson, OSUC 711133 (deposited in ANIC). *Paratypes*: **AUSTRALIA**: 31 females, OSUC 711124–711132, 711134–711149 (ANIC); OSUC 711150–711153 (CNCI); OSUC 711154–711155 (OSUC). *Other material*: **AUSTRALIA**: 1 female, USNMENT01197947 (ANIC).

#### Diagnosis.

*Calixomeria* possesses several autapomorphic characters that readily separate it from the rest of Sceliotrachelinae, the most salient of which are: LOL and OOL equal in length; knob of submarginal vein with a single, long, spine-like seta; the well-defined paracoxal sulcus; the large mesoscutellum, the posterior margin of which overhangs the metascutellum and most of the propodeum; and the presence of long, stout setae on tergites T2–T6.

In the key to world genera of Sceliotrachelinae (Masner and Huggert 1989), *Calixomeria* keys to couplet 31 separating *Helava* from *Alfredella* Masner & Huggert and *Aphanomerus* based on the pilosity of T1 and T2, and the presence or absence of foamy structures and median keels on the propodeum. *Calixomeria* lacks both setae that medially obscure the junction of T1 and T2 and foamy structures on the propodeum, thereby distinguishing it from *Helava*. Additionally, the propodeum is flat and lacks keels or protuberances, reliably separating *Calixomeria* from both *Alfredella* and *Aphanomerus*. The key of Masner and Huggert (1989) is modified to accommodate *Calixomeria*:

**Table:** 

31	Anterior margin of T2 densely setose; T1 with keel; propodeum with foamy structures (Fig. 19)	***Helava* Masner & Huggert**
–	Anterior margin of T2 glabrous or finely setose; T1 without keel; propodeum without foamy structures	**32**
32	Female antennae appearing 8-merous; A8–A10 cylindrical, subcompact (Fig. 20); posterior mesoscutellar sulcus clearly indicated (Fig. 20)	***Alfredella* Masner & Huggert**
–	Female antennae appearing 7-merous; A7–A10 ovoid, compact (Figs 1–3, 5, 7, 8); posterior mesoscutellar sulcus not defined	**32a**
32a	Mesoscutellum distinctly wider than long, not obscuring medial portion of propodeum in dorsal view (Fig. 22); transepisternal line present (Fig. 21); OOL less than 1 ocellar diameter from inner margin of compound eye (Fig. 22); propodeum with subparallel median keels or bulges (Fig. 22)	***Aphanomerus* Perkins**
–	Mesoscutellum approximately as wide as long, nearly as long as mesoscutum (Fig. 1); posterior portion of mesoscutellum obscuring medial portion of propodeum in dorsal view (Fig. 1); transepisternal line absent (Fig. 4); OOL more than 3 ocellar diameters from inner margin of compound eye (Fig. 1); propodeum without median keels or bulges (Figs 11, 15)	***Calixomeria* Lahey & Masner**

#### Discussion.

*Calixomeria* is a highly apomorphic genus within Sceliotrachelinae. In the generic cluster concepts of Masner and Huggert (1989), *Calixomeria* falls within the *Aphanomerus*-cluster due to its compact, ovoid clava with distinct sutures, a character shared with *Aphanomerella* Dodd, *Parabaeus* Kieffer, *Tetrabaeus* Kieffer, and some species of *Aphanomerus* Perkins (Fig. 11) and *Helava* Masner & Huggert (Talamas and Masner 2016). The remaining genera within the *Aphanomerus*-cluster have a subcompact (*Austromerus* Masner & Huggert and some *Helava*) or compact antennal clava without sutures (*Calomerella* Masner & Huggert, *Pseudaphanomerus* Szelényi, and most *Aphanomerus*).

Clavomeres are defined by the presence of papillary sensilla on the ventral surface of antennomeres of female platygastroids (Bin 1981). The apical four antennomeres (A7–A10) of *Calixomeria* females are enlarged, and A7 is fused to A8; however, A7 lacks papillary sensilla (Fig. 6). The only sceliotracheline hypothesized to have lost papillary sensilla on one or more of its antennomeres is *Pseudaphanomerus*, but in this genus sutures between the clavomeres are absent, resulting in a 1-merous clava with a claval formula of 3, presumably from the loss of papillary sensilla on A7 and A8 (Masner and Huggert 1989). *Aleyroctonus* Masner & Huggert may be another example of this reductive trend: its claval formula is 1-2-2 and A7 is enlarged relative to A6; however, A7 is clearly separated from the clava by a deep suture, which is faintly indicated in *Calixomeria*.

The elongate marginal cilia of the fore wing are found in relatively few taxa within Platygastroidea (e.g., *Dyscritobaeus* Perkins, *Embioctonus* Masner, *Encyrtoscelio* Dodd, *Eumicrosoma* Gahan, *Exon* Masner, *Idris* Förster). Sceliotrachelines that possess this character are *Errolium* Masner & Huggert and *Neobia* Masner & Huggert, but in these genera the apex of the submarginal vein nearly touches the anterior margin of the fore wing, whereas it is distant from the margin in *Calixomeria* (Fig. 3). In addition, there is a single, long, spine-like seta near the anterodorsal margin of the knob of the submarginal vein in *Calixomeria*, a character not known to us elsewhere in Platygastroidea.

*Calixomeria* is most easily recognized by its cup-shaped mesoscutellum that overhangs the metascutellum and most of the propodeum (Figs 1, 3, 4, 13). This character is not encountered elsewhere within Sceliotracheline, but is expressed to varying degrees in certain scelionids, some of which possess a mesoscutellum very similar in appearance to *Calixomeria* (e.g. *Gryon* Haliday). Convergence in the character systems mentioned above may reflect biological (i.e., host choice) or environmental (i.e., habitat) similarities between these genera. Unfortunately, host associations are known for just a fraction of the superfamily. Molecular data from additional, freshly collected specimens would greatly facilitate the placement of this taxon within the framework of Sceliotrachelinae.

## Supplementary Material

XML Treatment for Calixomeria lasallei
